# Tregs Promote Astrocyte‐Neuron Lactate Shuttle via Inhibiting STING Pathway to Improve Neurological Recovery After Ischemic Stroke

**DOI:** 10.1002/cns.70753

**Published:** 2026-01-19

**Authors:** Yao Meng, Xiaoyan Li, Yonghong Bi, Pengyu Duan, Zhehao Jin, Lan Luo, Weiyu Feng, Hangbing Li, Xiangcheng Zhao, Kun Zuo, Jiali Chen, Longfei Li, Yuling Xing, Miao Yu, Muyan Cui, Yang Yu, Bing Zhang

**Affiliations:** ^1^ Department of Anesthesiology The Second Affiliated Hospital of Harbin Medical University Harbin China

**Keywords:** astrocyte‐neuron lactate shuttle, ischemic stroke, neurological recovery, STING, Tregs

## Abstract

**Background:**

Excessive immune response following ischemic stroke is closely associated with poor clinical prognosis. Although regulatory T cell (Treg) is recognized as pivotal immunomodulator, its potential mechanisms in post‐stroke neurological recovery and immunotherapy remain unclear.

**Methods:**

Neurological recovery and neuronal remodeling were investigated by behavior tests, HE staining, Nissl staining, and LFB staining. Monocarboxylate transporter (MCT) was detected to evaluate the role of astrocyte‐neuron lactate shuttle (ANLS) in Tregs‐mediated neuroprotection by immunofluorescence and western blot, lactate assay, ATP assay, and cell viability assay experiments. The expression of stimulator of interferon gene (STING) and phosphorylation of downstream factors were examined by western blot.

**Results:**

Tregs significantly attenuated neuronal injury, upregulated the expression of synaptic plasticity‐related proteins, promoted myelin reconstruction, and improved spatial cognition, memory function, and motor coordination. Mechanistically, Tregs enhanced MCT‐mediated lactate transfer to neurons, providing energy supply for neuronal remodeling, whereas the MCT inhibitor 4‐CIN reversed Tregs‐mediated neuroprotection. In addition, Tregs suppressed the activation of the STING pathway, and activation of STING by DMXAA abolished the Tregs‐induced potentiation of ANLS.

**Conclusions:**

This study clarifies a novel mechanism by which Tregs promote ANLS and provide energy supply for neuronal remodeling by inhibiting the STING pathway, thereby improving the long‐term neurological recovery after stroke.

## Graphical abstract

Tregs improve long‐term neurological recovery after ischemic stroke. Tregs promote ANLS and provide energy supply for neuronal remodeling after stroke. The mechanism underlying Tregs‐mediated ANLS enhancement involves suppression of the STING pathway.

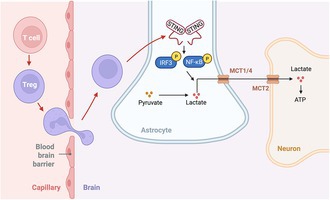

**Abbreviations**
4‐cin: α‐cyano‐4‐hydroxycinnamic acid
ANLS: Astrocyte‐neuron lactate shuttle
DMXAA: Vadimezan
Foxp3: Transcription factor forkhead box P3
IL‐10: Interleukin‐10
IRF3: Interferon regulatory factor 3
MCAO: Middle cerebral artery occlusion
MCT: Monocarboxylate transporter
NF‐κB: Nuclear factor kappa‐B
OGD/R: Oxygen–glucose deprivation/reoxygenation
STING: Stimulator of interferon gene
TGF‐β: Transforming growth factor‐β
Treg: Regulatory T cell

## 1 Background

Ischemic stroke is a worldwide disease with high mortality and disability rates, which not only affects the quality of life of patients, but also brings a heavy economic burden to families and society [1]. The interruptions of cerebral blood supply after ischemic stroke can lead to impaired sensory, motor, and memory function, resulting in a poor prognosis [2]. Tissue‐type plasminogen activator (t‐PA) is currently considered the only effective treatment for ischemic stroke, but the therapeutic time window of t‐PA is limited, and only a small number of patients can benefit from it [3]. The mechanisms contributing to neurological impairment after stroke are complex, and the crosstalk between the central nervous system and the immune system, as well as the activation and recruitment of immune cells, are critical for inhibiting inflammation and neurological recovery after stroke [4, 5]. However, excessive immune responses can impair the self‐repair capacity of brain tissue and exacerbate brain damage [6]. Thus, maintaining immune homeostasis is of great significance for improving long‐term neurological recovery after ischemic stroke.

Regulatory T cell (Treg) is a subpopulation of CD4 characterized as CD4^+^CD25^+^, and transcription factor forkhead box P3 (Foxp3) [7]. Treg is a key target of immunomodulation and plays an important role in the restoration of immune homeostasis and neuronal remodeling after ischemic stroke [8]. Tregs inhibit systemic inflammatory immune responses and peripheral effector T cell activation by secretion of immunosuppressive cytokines such as interleukin‐10 (IL‐10) and transforming growth factor‐β (TGF‐β) [9]. Besides, Tregs can promote neurological recovery after stroke by alleviating the damage to the blood–brain barrier integrity and inhibiting astrogliosis [10, 11]. Current research of Tregs on ischemic stroke mainly focuses on the acute and the subacute stages after stroke. Whether and how Tregs affect the recovery of brain function in the remote stage of stroke remain to be explored in depth.

Brain activity and neuronal remodeling require sufficient energy supply [12, 13]. Lactate has long been considered as a metabolic waste and a marker of tissue suffering. However, recent studies have confirmed that lactate is also a crucial energy substrate in the central nervous system, which can replace glucose under pathological conditions to provide energy support for neuronal repair and remodeling [14], and participate in brain activity as a signaling molecule [15, 16]. Astrocyte‐neuron lactate shuttle (ANLS) is a vital model of lactate transmission, which mainly involves astrocytes producing lactate in response to neurons and delivering it to neurons via monocarboxylate transporter (MCT) to satisfy neuronal energy needs [17]. Investigations have demonstrated that ANLS exerts beneficial effects in preventing neuronal damage and cognitive dysfunction through providing energy supply [18]. Taken together, post‐stroke neuronal remodeling requires sustained energy supply, ANLS is an important pathway for maintaining neuronal energy homeostasis after stroke, and Tregs are closely related to the recovery of neurological function after stroke, so whether Tregs can improve post‐stroke neurological regression through ANLS arouses our interest.

Stimulator of interferon gene (STING) is a key participant in the innate immune response which is sensed by cyclic GMP–AMP synthase (cGAS) and other DNA sensors to activate interferon regulatory factor 3 (IRF3) and nuclear factor kappa‐B (NF‐κB) to drive inflammatory disease [19, 20]. STING plays a vital role in Tregs‐driven immune cascade by regulating various chemokines or cytokines in the microenvironment [21, 22]. In addition, studies have confirmed that STING is involved in the pyroptosis and senescence of astrocytes [23, 24]. However, it is unclear whether STING is associated with ANLS. In this study, we aim to investigate whether enhancing the number of Tregs through the administration of IL‐2:IL‐2 antibody complex (IL‐2:IL‐2 Ab) could improve long‐term neurological recovery after ischemic stroke and the underlying mechanisms, thereby providing novel targets and innovative solutions for stroke treatment.

## 2 Materials and Methods

### 2.1 Animals

Male C57BL/6 mice were purchased from the Animal Experiment Center at the Second Affiliated of Harbin Medical University. All mice were housed in a specific‐pathogen‐free (SPF) environment with a consistent 12 h light/12 h dark cycle and were given *ad libitum* access to food and water. Animal experimental protocols were conducted following the National Institutes of Health guidelines (Guide for the Care and Use of Laboratory Animals) and were approved by the Animal Ethics Committee of the Second Affiliated Hospital of Harbin Medical University (YJSDW2024‐009).

### 2.2 Murine Model of Ischemic Stroke

In this study, 8–10‐week‐old male mice weighing 20–25 g were used to establish the middle cerebral artery occlusion (MCAO) model. After anesthetizing with sevoflurane (3% induction and 1.5% maintenance with spontaneous respiration, in 30% O_2_), a 6–0 silicone‐coated suture (Doccol Corporation, MA, USA) was carefully inserted into the left internal carotid artery until reaching the origin of the MCA. After 90 min of occlusion, the filament was removed to initiate cerebral blood reperfusion. Sham procedures followed similar steps but omitted the insertion of sutures. Body temperature of mice was maintained at 37.5°C ± 0.5°C using a controlled heating system throughout all procedures.

### 2.3 Intraperitoneal Injection of IL‐2:IL‐2 Antibody Complex

IL‐2 protein (575,406, BioLegend, USA) was mixed with anti‐IL‐2 Ab (503706, BioLegend, USA) at a 2:1 molar ratio (1 μg of recombinant murine IL‐2 protein and 5 μg of anti‐IL‐2 Abs) and incubated at 37°C for 30 min [25]. IL‐2:IL‐2 Ab complex or IgG isotype control (405414, BioLegend, USA) was intraperitoneally injected into mice once per day for 3 consecutive days starting 6 h after MCAO and repeated once per day on day 10 and 20 after MCAO [26].

### 2.4 Drug Administration

α‐cyano‐4‐hydroxycinnamic acid (4‐cin, an MCT inhibitor, MedChemExpress) and Vadimezan (DMXAA, a STING agonist, MedChemExpress) were freshly dissolved in 1.5% dimethyl sulfoxide (DMSO). 4‐cin (100 mg/kg) [27], DMXAA (15 mg/kg) [28] or vehicle was intraperitoneally administered once per 2 days from 24 h after reperfusion to euthanized.

### 2.5 Treg Cell Isolation

Single cell suspensions were prepared from the spleens of mice (8–10‐week‐old). CD4^+^CD25^+^ Treg cells were isolated using a mouse Treg cell isolation kit (130‐091‐041, Miltenyi Biotec, Germany) according to the manufacturer's instructions. The isolation was performed in a two‐step procedure with a negative selection for CD4^+^ cells and a positive selection for CD25^+^ cells.

### 2.6 Cell Culture and Treatment

Mice astrocyte type I clone (C8‐D1A) and hippocampal neuron (HT22) were obtained from Procell (Wuhan, China), and cultured in Dulbecco's Modified Eagle's Medium (DMEM) supplemented with 10% fetal bovine serum (FBS) and 1% penicillin/streptomycin at 37°C in a 5% CO_2_ stable environment. Treg cells were cultured in Roswell Park Memorial Institute (RPMI)‐1640 Medium supplemented with 10% FBS, 1% penicillin/streptomycin, 1% GlutaMax and stimulated with anti‐CD3 (4 μg/mL, eBioscience, USA), anti‐CD28 (5 μg/mL, eBioscience, USA), and IL‐2 (100 ng/mL).

The C8‐D1A cells were treated with 4‐cin (30 μg/mL) [29] or DMXAA (75 μg/mL) [30]. Cells were transfected with si‐STING and si‐NC (QE gene, China) and then cultured in normal medium for 24 h for subsequent experiments.

### 2.7 Oxygen–Glucose Deprivation/Reoxygenation (OGD/R)

C8‐D1A cells were transitioned to deoxygenated glucose‐free DMEM and then incubated in an anaerobic chamber filled with a gas mixture of 5% CO_2_ and 95% N_2_ for a period of 2 h at 37°C. Then, the cells were returned to normal culture conditions with DMEM supplemented with 10% FBS and glucose, incubated at 37°C in a humidified atmosphere of 5% CO_2_ for an additional 24 h.

The cell culture supernatant of C8‐D1A cells was collected and centrifuged at 1000 rpm for 5 min to remove cell debris. The supernatant was then filtered through a 0.22 μm filter to obtain medium for HT22 cells OGD/R modeling.

### 2.8 Transwell

C8‐D1A cells were seeded on the apical chamber of the transwell, and isolated Tregs were seeded on the bottom chamber of the transwell in the presence of soluble anti‐CD3, anti‐CD28, and IL‐2 for 1 day.

### 2.9 TTC Staining

Mice were euthanized and brain tissues were collected 24 h after reperfusion. The brains were frozen at −20°C for 15 min, sectioned into approximately 1‐mm‐thick slices, and stained with 2% TTC solution at 37°C for 20 min. Infarcted regions appeared white, whereas non‐infarcted regions were stained red.

### 2.10 Flow Cytometry

Mice were deeply anesthetized and euthanized to separate brain tissues at 14 days after reperfusion. Brain tissues were grounded into single cell suspension to perform flow staining. Single cell samples were first incubated with surface antigens anti‐mouse‐CD4, PE/Cyanine7 (100421, BioLegend, USA) and anti‐mouse‐CD25, APC (102011, BioLegend, USA) for 30 min on ice at 4°C in the dark. Cells were fixed and permeabilized by Fixation/Permeabilization Diluent & Concentrate according to the manufacturer's instructions, followed by anti‐mouse‐Foxp3, Brilliant Violet 421 (126419, BioLegend, USA) incubation recommended concentration for intracellular staining for 30 min. Flow cytometry was performed on the NovoCyte 3110 (Agilent, USA). Data analyses were performed using FlowJo software.

### 2.11 Rotarod Test

The rotarod test was used to assess motor coordination, which mice were forced to run on a rotating drum (SA102/LF072003, SansBio) [31]. The mice were trained at a constant speed of 20 rpm for three sessions on three consecutive days before testing. During the test, the rotarod speed was increased from 0 to 40 rpm within 300 s. The latency to fall off the drum was recorded automatically for each mouse, with an interval of 15 min. Data were expressed as mean values from three trials per day.

### 2.12 Morris Water Maze

The Morris water maze (MWM) was used to assess spatial cognitive and memory function [32]. The experiment consists of two phases: learning and memory. The learning test was carried out 22–26 days after reperfusion and the time spent to reach the platform was recorded as the escape latency to reflect spatial cognitive function. In the memory test, the platform was removed and a single 60 s probe trial for mice to swim freely was conducted. Time spent in the goal quadrant and swimming speed were recorded to reflect spatial memory function. An automated tracking system was used to record and analyze behavioral data.

### 2.13 Hematoxylin and Eosin (HE) Staining

Mice were deeply anesthetized and transcardially perfused with 20 mL PBS followed by 20 mL of 4% paraformaldehyde (PFA) at 21 days after reperfusion. Brain tissues were separated and post‐fixed in PFA overnight at 4°C, embedded in paraffin wax. Brain tissues were cut into 5 μm sections and treated with hematoxylin and eosin, followed by imaging under a microscope.

### 2.14 LFB Staining

Luxol fast blue (LFB) staining was used to demonstrate myelination [33]. Hippocampal 5 μm paraffin coronal sections were treated with LFB myelin stain kit (Solarbio, China) according to the manufacturer's instructions. Finally, the sections were imaged under a microscope.

### 2.15 Nissl Staining

Paraffin coronal brain tissue sections were treated with Nissl staining solution (Solarbio, China) according to the manufacturer's instructions. Three stained sections were randomly selected from each experimental mouse to evaluate the average number of Nissl positive cells.

### 2.16 Immunofluorescence

Tissues and cells were incubated with primary antibodies against MCT4 (1:500, ab308528, Abcam), STING (1:200, ab288157, Abcam), GFAP (1:500, DF6040, Affinity), MCT2 (1:200, 20335‐1‐AP, Proteintech) and Neun (1:500, DF6145, Affinity) for staining overnight at 4°C and washed three times with PBS. Then, the tissues were incubated with the secondary antibodies at room temperature for 1 h in the dark, washed three times with PBS. Finally, the nuclei were counterstained with DAPI (Beyotime, China) at room temperature for 10 min in the dark, washed with PBS for 10 min and sealed after drying. Images were captured using a fluorescence microscope (Zeiss, Germany).

### 2.17 Enzyme‐Linked Immunosorbent Assay (ELISA)

The hippocampus was rapidly collected and stored at −80°C at 21 days after reperfusion. One gram of tissue was fully homogenized with 9 mL PBS, followed by centrifuging at 3000 rpm at 4°C for 20 min, and the supernatant was collected for ELISA. The expression levels of IL‐10 and TGF‐β in the hippocampus were detected by ELISA kit (Jingmei, China) following the manufacturer's instructions. Protein concentration in the supernatant was quantified using BCA assay. The absorbance was measured with a microplate reader at 450 nm.

### 2.18 Western Blot

The hippocampus of mice and cells was rapidly collected and stored at −80°C. Samples were homogenized, lysed, and quantified using BCA assay, with the loading volume adjusted for subsequent electrophoresis. Polyvinylidene difluoride membranes (PVDF, Millipore, USA) were blocked with 5% skim milk and incubated overnight at 4°C with the following primary antibodies: Foxp3 (1:1000, ab215206, Abcam), PSD95 (1:1000, AF5283, Affinity), GAP43 (1:1000, DF7766, Affinity), Synaptophysin (1:1000, WL03058, Wanleibio), MCT4 (1:1000, ab308528, Abcam), MCT2 (1:1000, 20335–1‐AP, Proteintech), STING (1:1000, YT5488, Immunoway), IRF3 (1:1000, YT2396, Immunoway), p‐IRF3 (1:1000, YP0880, Immunoway), NF‐κB p65 (1:1000, 8242S, Cell Signaling Technology), p‐NF‐κB p65 (1:1000, 3033S, Cell Signaling Technology), NLRP3 (1:1000, ab263899, Abcam), GSDMD‐N (1:1000, DF13758, Affinity) and β‐actin (1:10000, AC050, ABclonal). After incubation, the membranes were incubated with HRP‐conjugated Goat anti‐Rabbit IgG (1:5000, ZSBIO, China) for 1 h at room temperature. Finally, the bands were detected with an enhanced chemiluminescent ECL (Biosharp, Beijing, China). Blot images were captured with a ChemiDoc touch imaging system. The bands were quantified using Image J software.

### 2.19 Cell Viability Assay

Cell viability was detected using cell counting kit‐8 (CCK‐8, Adamas life, China). Transferred 100 μL cell suspensions to a 96‐well plate for different treatments, and then incubated with 10 μL of CCK‐8 solution at 37°C/5% CO_2_ for 2 h. The absorbance was measured at 450 nm with a microplate reader.

### 2.20 Lactate Dehydrogenase (LDH) Assay

The cell culture supernatants were collected, and the quantity of LDH in the supernatants was determined using a lactate dehydrogenase (LDH) activity assay kit (Solarbio, China) according to the manufacturer's instructions. The absorbance was measured at 450 nm with a microplate reader.

### 2.21 Lactate Assay

The cell suspensions were collected, and the quantity of lactate in the cells was determined using a L‐lactate (L‐LA) content assay kit (Solarbio, China) according to the manufacturer's instructions. The absorbance was measured at 570 nm with a microplate reader.

### 2.22 ATP Assay

The cell suspensions were collected, and the quantity of ATP was determined using an ATP content assay kit (Solarbio, China) according to the manufacturer's instructions. The absorbance was measured at 340 nm with a microplate reader.

### 2.23 Statistical Analysis

Data were presented as mean ± standard deviation (SD) and analyzed and plotted of statistical graphs using the software of GraphPad Prism 10.0 and Image J. The Shapiro–Wilk normality test was used to test the normality of the continuous data. Statistical significance was performed using Student's *t*‐test, one‐way analysis of variance (ANOVA) with Turkey's multiple comparison test. A repeated measures analysis of ANOVA was used to compare escape latency in the Morris water maze and rotarod test in different groups. A value of *p* < 0.05 was considered statistically significant.

## 3 Results

### 3.1 Increasing the Number of Tregs Improved Long‐Term Neurological Recovery After Stroke in Mice

**FIGURE 1:**
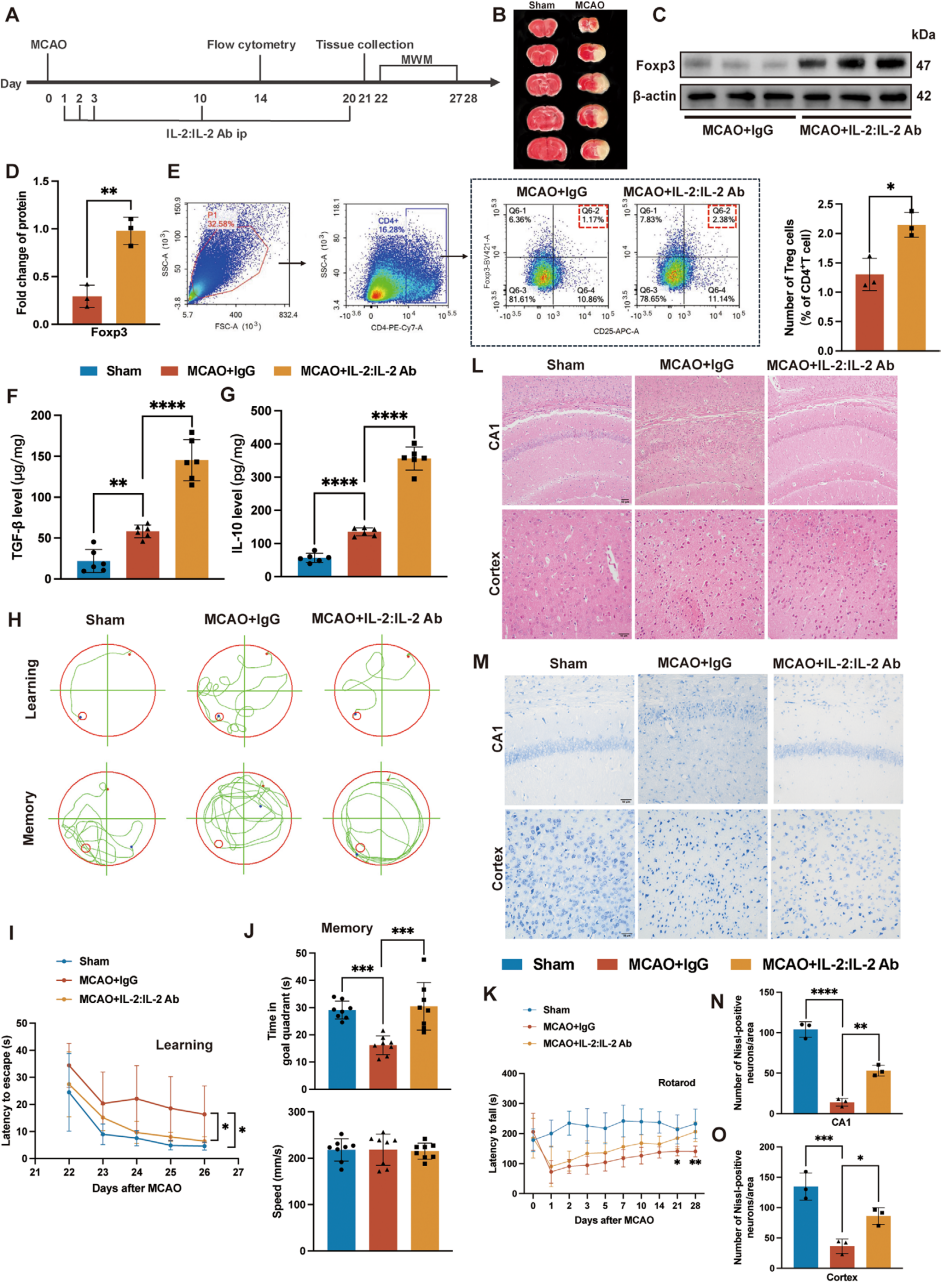
Increasing the number of Tregs improved long‐term neurological recovery after stroke in mice. (A) Overview of the experimental flowchart. (B) TTC staining images after MCAO operation. (C) Representative bands of protein Foxp3 in mouse brains. (D) Quantification of the relative protein expression of Foxp3 (*n* = 3). (E) Flow cytometry analysis of CD4^+^CD25^+^Foxp3^+^ Tregs in brains on 14 days after stroke (*n* = 3). (F–G) The levels of TGF‐β (F_2,15_ = 81.26, *p* < 0.0001) and IL‐10 (F_2,15_ = 282.7, *p* < 0.0001) in the hippocampus were quantified by ELISA (*n* = 6). (H) Spatial cognitive and memory functions were evaluated in the Morris water maze. Representative swimming trajectories in the Morris water maze. (I) The escape latency was recorded for 5 consecutive days (*n* = 8). (J) Time (F_2,21_ = 15.11, *p* < 0.0001) and speed (F_2,21_ = 0.03499, *p* = 0.9657) spent in the target quadrant (*n* = 8). (K) Motor coordination function was assessed by the rotarod test (*n* = 8). (L) HE staining of hippocampal CA1 region and cortex. Scale bar = 50 μm. (M‐O) Nissl staining of hippocampal CA1 region (F_2,6_ = 113.1, *p* < 0.0001) and cortex (F_2,6_ = 26.17, *p* = 0.0011) (*n* = 3). Scale bar = 50 μm. Quantitative data were shown as mean ± SD. *p < 0.05, **p < 0.01, ***p < 0.001, ****p < 0.0001.

The experimental flowchart for in vivo study was illustrated in Figure 1A. The establishment of the MCAO model was confirmed by TTC staining (Figure 1B). Western blot analysis revealed that Foxp3 protein levels were elevated in the MCAO + IL‐2:IL‐2 Ab group relative to the MCAO + IgG group (Figure 1C,D). Flow cytometry analysis showed that compared with the MCAO + IgG group, the ratio of CD4^+^CD25^+^Foxp3^+^ Tregs increased significantly in the MCAO + IL‐2:IL‐2 Ab group on the 14th day after stroke (Figure 1E). In addition, the levels of TGF‐β and IL‐10, the downstream activation factors of Tregs, were higher in the MCAO + IL‐2:IL‐2 Ab group than those of the MCAO + IgG group (Figure 1F,G). The above results showed that the quantity and function of Tregs increased after administration of the IL‐2:IL‐2 Ab complex.

In the Morris water maze assay, the latency to find the target platform was significantly prolonged in the MCAO + IgG group when compared with the Sham group, while IL‐2:IL‐2 Ab complex treatment attenuated this impairment (Figure 1H,I). In the memory stage, the experimental results revealed that mice in the MCAO + IL‐2:IL‐2 Ab group stayed longer in the target zone compared with the MCAO + IgG group, suggesting that Tregs could alleviate spatial memory impairment caused by stroke. There was no significant difference in swimming speed between different groups, indicating that swimming skills were similar between groups (Figure 1J). Motor coordination function was measured by the rotarod test, and it was found that IL‐2:IL‐2 Ab complex‐treated mice had improved motor coordination function about 21 days after MCAO compared with the MCAO + IgG group (Figure 1K).

HE staining showed that compared with the Sham group, the MCAO + IgG group exhibited significant tissue disorganization, nuclear consolidation, and gliocytosis in the hippocampal CA1 region and cortex, while the tissue disturbance was reduced in the MCAO + IL‐2:IL‐2 Ab group (Figure 1L). Nissl staining indicated a significant increase in neuron survival in the hippocampal CA1 region and cortex in the IL‐2:IL‐2 Ab complex‐treated group compared with the MCAO + IgG group (Figure 1M–O). These results demonstrated that IL‐2:IL‐2 Ab complex increased the number and function of Tregs, mitigated cognitive and motor functions, reduced neuronal damage, and improved long‐term neurological recovery after stroke.

### 3.2 Tregs Enhanced ANLS After Stroke in Mice

**FIGURE 2:**
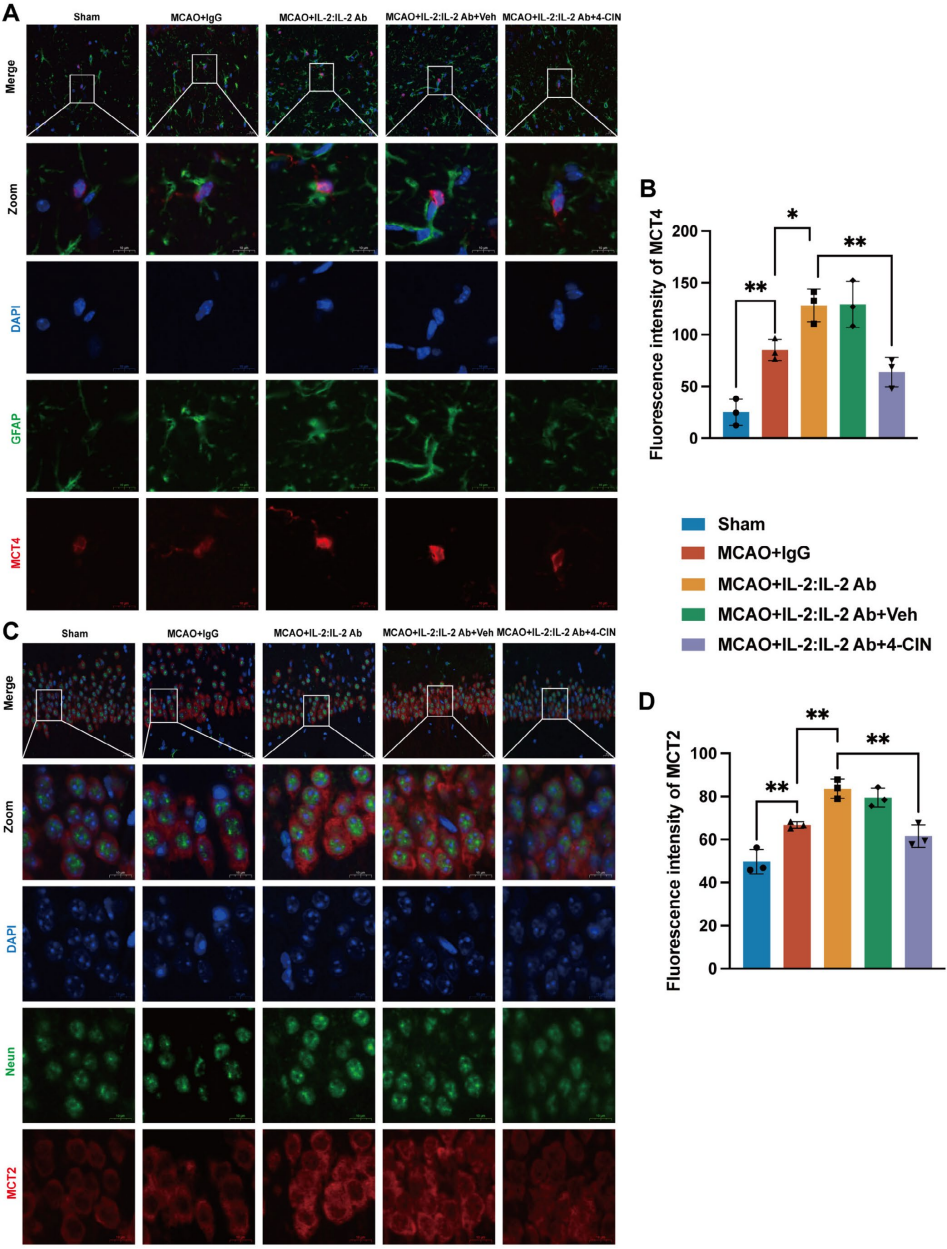
Tregs enhanced ANLS after stroke in mice. Representative images and fluorescence intensity of MCT4 (A–B: F_4,10_ = 24.19, *p* < 0.0001) in astrocytes and MCT2 (C–D: F_4,10_ = 27.36, *p* < 0.0001) in neurons immunofluorescence in the hippocampal CA1 region (*n* = 3). Scale bars = 20 μm and 10 μm. Quantitative data are shown as mean ± SD. *p < 0.05, **p < 0.01.

ANLS was evaluated with MCT expression. MCT4‐positive cells were highly co‐localized with astrocytes, and MCT2‐positive cells were highly co‐localized with neurons in the hippocampal CA1 region (Figure 2A,C). In addition, the immunofluorescence results revealed that IL‐2:IL‐2 Ab complex significantly increased the number of MCT proteins, whereas 4‐CIN reversed the promoting effect of Tregs on ANLS (Figure 2B,D). These findings indicated that Tregs effectively enhanced ANLS after stroke in mice.

### 3.3 Inhibition of ANLS by 4‐CIN Suppressed the Protective Effect of Tregs on Long‐Term Neurological Recovery and Neuronal Remodeling After Stroke in Mice

**FIGURE 3:**
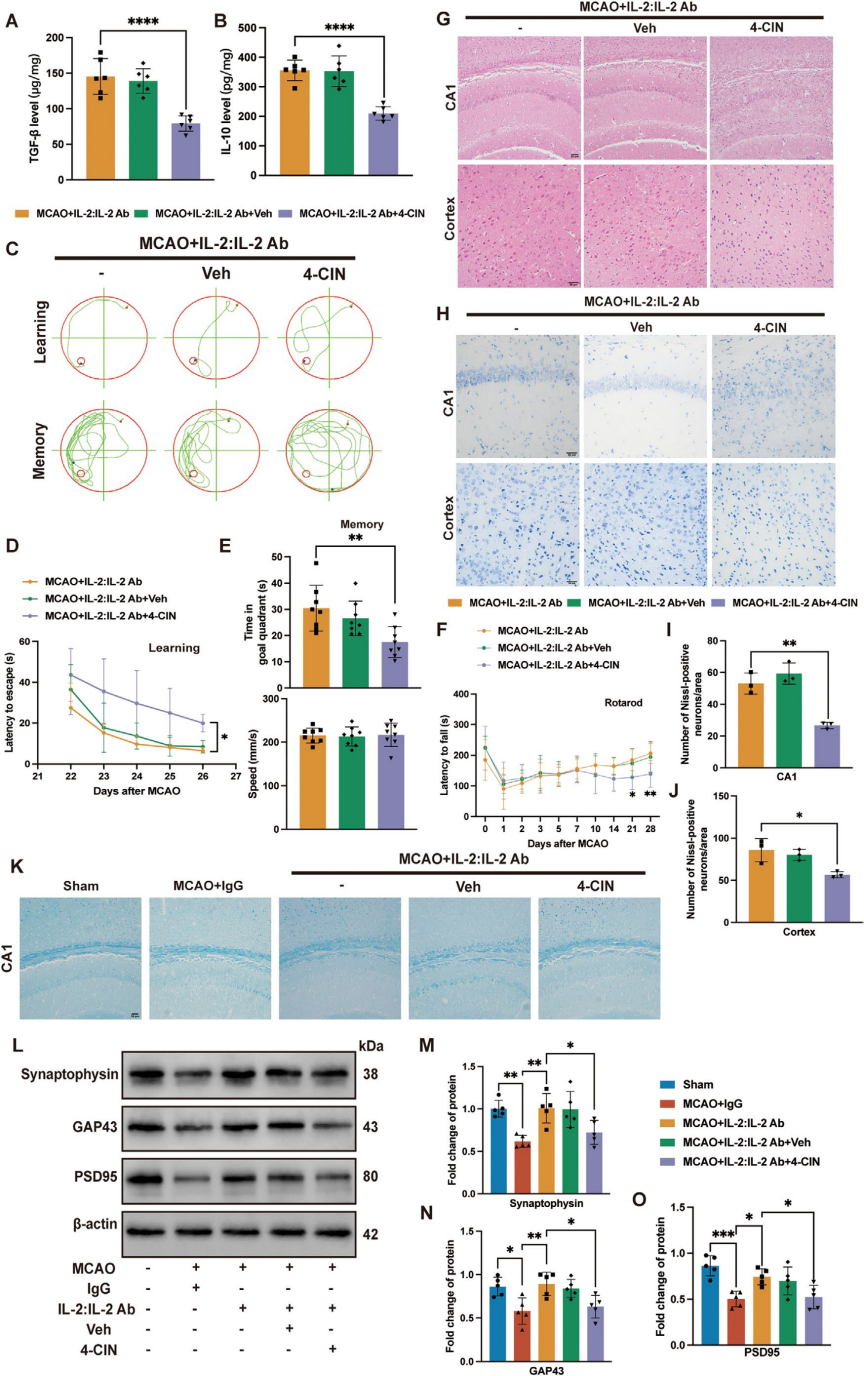
Inhibition of ANLS by 4‐CIN suppressed the protective effect of Tregs on long‐term neurological recovery and neuronal remodeling after stroke in mice. (A–B) The levels of TGF‐β (F_2,15_ = 22.87, *p* < 0.0001) and IL‐10 (F_2,15_ = 28.39, *p* < 0.0001) in the hippocampus were quantified by ELISA (*n* = 6). (C) Spatial cognitive and memory functions were evaluated in the Morris water maze. Representative swimming trajectories in the Morris water maze. (D) The escape latency was recorded for 5 consecutive days (*n* = 8). (E) Time (F_2,21_ = 6.922, *p* = 0.0049) and speed (F_2,21_ = 0.05306, *p* = 0.9485) spent in the target quadrant (*n* = 8). (F) Motor coordination function was assessed by the rotarod test (*n* = 8). (G) HE staining of hippocampal CA1 region and cortex. Scale bar = 50 μm. (H–J) Nissl staining of hippocampal CA1 region (F_2,6_ = 29.21, *p* = 0.0008) and cortex (F_2,6_ = 8.907, *p* = 0.0160) (*n* = 3). Scale bar = 50 μm. (K) LFB staining of hippocampal CA1 region. Scale bar = 50 μm. (L) Representative bands of synaptic plasticity‐associated proteins Synaptophysin, GAP43 and PSD95 in the hippocampus. (M‐O) Quantification of the relative protein expression of Synaptophysin (F_4,20_ = 7.844, *p* = 0.0006), GAP43 (F_4,20_ = 6.566, *p* = 0.0015) and PSD95 (F_4,20_ = 8.922, *p* = 0.0003) (*n* = 5). Quantitative data were shown as mean ± SD. *p < 0.05, **p < 0.01, ***p < 0.001, ****p < 0.0001.

To determine whether ANLS is involved in the excitation and neuroprotection effects of Tregs, we used 4‐CIN on the basis of increasing the number of Tregs. 4‐CIN significantly decreased TGF‐β and IL‐10 levels in the hippocampus compared with the MCAO + IL‐2:IL‐2 Ab group (Figure 3A,B). The improved effects of Tregs on spatial cognition (Figure 3C,D), spatial memory (Figure 3E), and motor coordination function (Figure 3F) were reversed after using the MCT inhibitor 4‐CIN. In addition, HE staining showed that compared with the MCAO + IL‐2:IL‐2 Ab group, the MCAO + IL‐2:IL‐2 Ab + 4‐CIN group exhibited more disorganization, nuclear consolidation and gliocytosis in the hippocampal CA1 region and cortex (Figure 3G). Nissl staining indicated a significant decrease in neuron survival in the hippocampal CA1 region and cortex in the MCAO + IL‐2:IL‐2 Ab+4‐CIN group (Figure 3H–J). Furthermore, LFB staining revealed that the myelin structure in the hippocampal CA1 region was incomplete with loose arrangement and obvious fracture following stroke. Treatment with IL‐2:IL‐2 Ab complex significantly improved the myelin structure, suggesting enhanced regenerative ability. Conversely, administration of 4‐CIN resulted in destruction of the myelin structure, indicating impaired regenerative ability (Figure 3K). In addition, the expression of synaptic plasticity‐associated proteins Synaptophysin, PSD95 and GAP43 in the hippocampus was significantly decreased following stroke, which were upregulated by IL‐2:IL‐2 Ab complex and inhibited by 4‐CIN (Figure 3L–O). These results confirmed that the role of Tregs in improving neurological recovery and neuronal remodeling after stroke was achieved by promoting ANLS.

### 3.4 Tregs Attenuated HT22 Injury Through Promoting ANLS After OGD/R In Vitro

**FIGURE 4:**
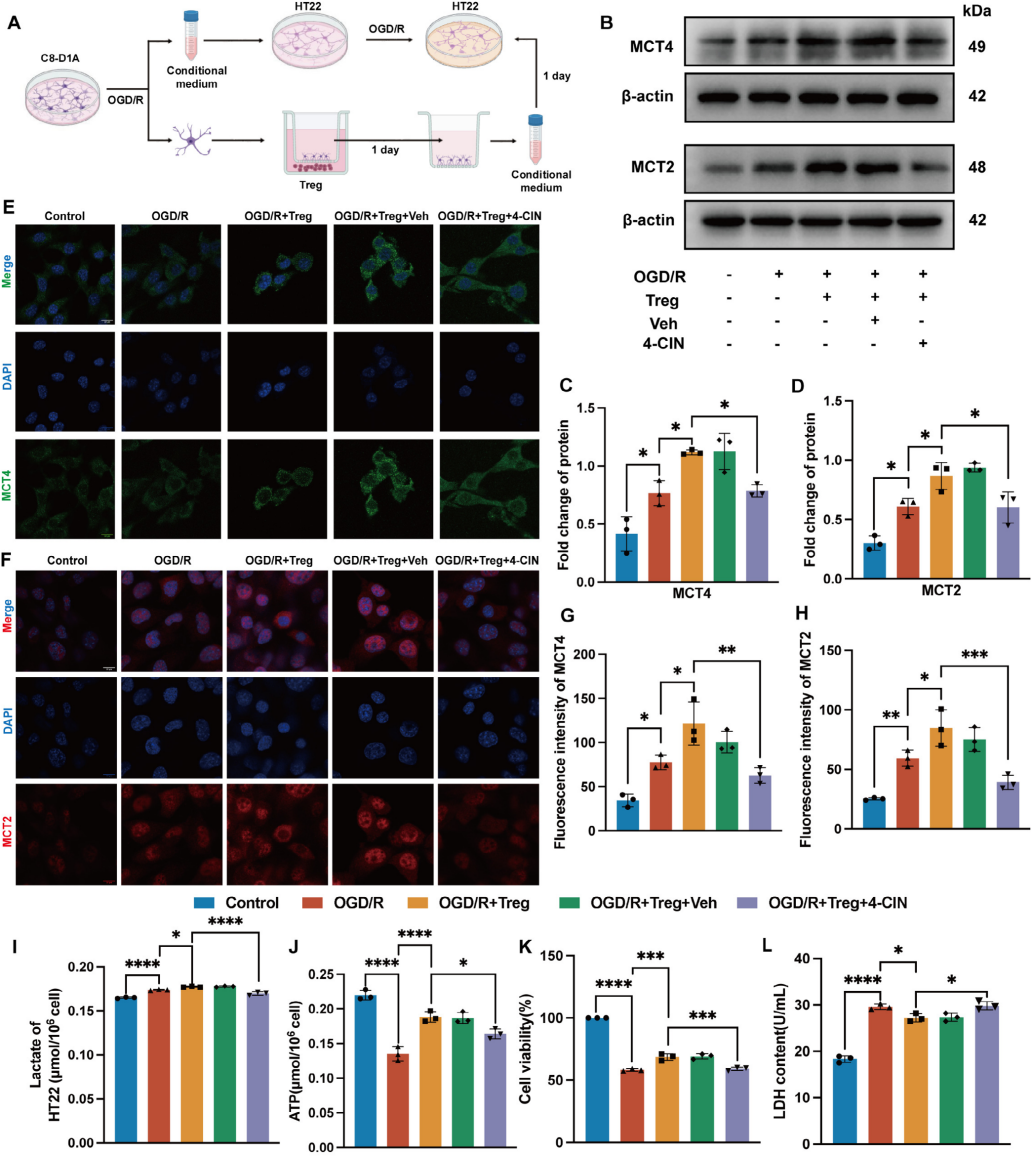
Tregs attenuated HT22 injury through promoting ANLS after OGD/R in vitro. (A) C8‐D1A cells were seeded in cell culture dish for OGD/R and then co‐cultured with Tregs for 1 day. The culture of C8‐D1A cells at different stages was collected as conditional medium for HT22 cells. (B) Representative bands of ANLS‐related proteins MCT4 of C8‐D1A cells, MCT2 of HT22 cells. (C–D) Quantification of the relative protein expression of MCT4 (F_4,10_ = 21.14, *p* < 0.0001) and MCT2 (F_4,10_ = 23.98, *p* < 0.0001) (*n* = 3). (E‐H) Immunofluorescence staining images and quantitative analysis of MCT4 (F_4,10_ = 17.89, *p* = 0.0001) in C8‐D1A cells, MCT2 (F_4,10_ = 21.70, *p* < 0.0001) in HT22 cells (*n* = 3). Scale bars = 10 μm. (I) Lactate level of HT22 cells (F_4,10_ = 77.72, *p* < 0.0001) (*n* = 3). (J) ATP level of HT22 cells (F_4,10_ = 46.24, *p* < 0.0001) (*n* = 3). (K) CCK‐8 was used to evaluate cell viability of HT22 cells in different groups (F_4,10_ = 298.8, *p* < 0.0001) (*n* = 3). (L) LDH level of HT22 cells supernatant (F_4,10_ = 103.5, *p* < 0.0001) (*n* = 3). Quantitative data were shown as mean ± SD. *p < 0.05, **p < 0.01, ***p < 0.001, ****p < 0.0001.

Based on the above results, C8‐D1A cells, HT22 cells, and isolated Tregs were selected for further in vitro experiment. The C8‐D1A cells were subjected to OGD/R, followed by collecting their conditioned medium for co‐culture with HT22 cells to mimic a stroke‐associated cerebral environment. After OGD/R treatment, C8‐D1A cells interfered with Tregs, followed by collecting their conditioned medium for co‐culture with HT22 cells after OGD/R to mimic the Tregs‐treated cerebral environment (Figure 4A). The purity of isolated Tregs was confirmed by flow cytometry (Figure S1A). The survival rate of C8‐D1A cells after co‐culture with different amounts of Tregs was determined by CCK‐8, and the results showed that the optimal co‐culture ratio was C8‐D1A cells: Tregs = 1:10 (Figure S1B).

To investigate the effect of Tregs on ANLS, we further detected the expression of MCT and lactate in cells. Western blot analysis revealed that Tregs increased the protein expression of MCT4 and MCT2, but 4‐CIN inhibited the effect of Tregs (Figure 4B–D). Immunofluorescence staining of MCT4 and MCT2 confirmed an increase in the immunofluorescence intensity of MCT4 and MCT2 following co‐culture with Tregs, which was reversed by 4‐CIN (Figure 4E–H).

Moreover, compared with the OGD/R group, the lactate and ATP content were significantly increased in Treg‐treated HT22 cells, indicating an increase in lactate transport levels and energy supply, while 4‐CIN inhibited this effect (Figure 4I,J). Furthermore, the results of CCK‐8 and LDH assay showed that 4‐CIN inhibited the protective effect of Tregs on HT22 cells (Figure 4K,L). Taken together, these results suggested that the therapeutic effect of Tregs was mediated by promoting ANLS.

### 3.5 Activation of STING by DMXAA Inhibited the Enhancement of ANLS by Tregs in Mice

**FIGURE 5:**
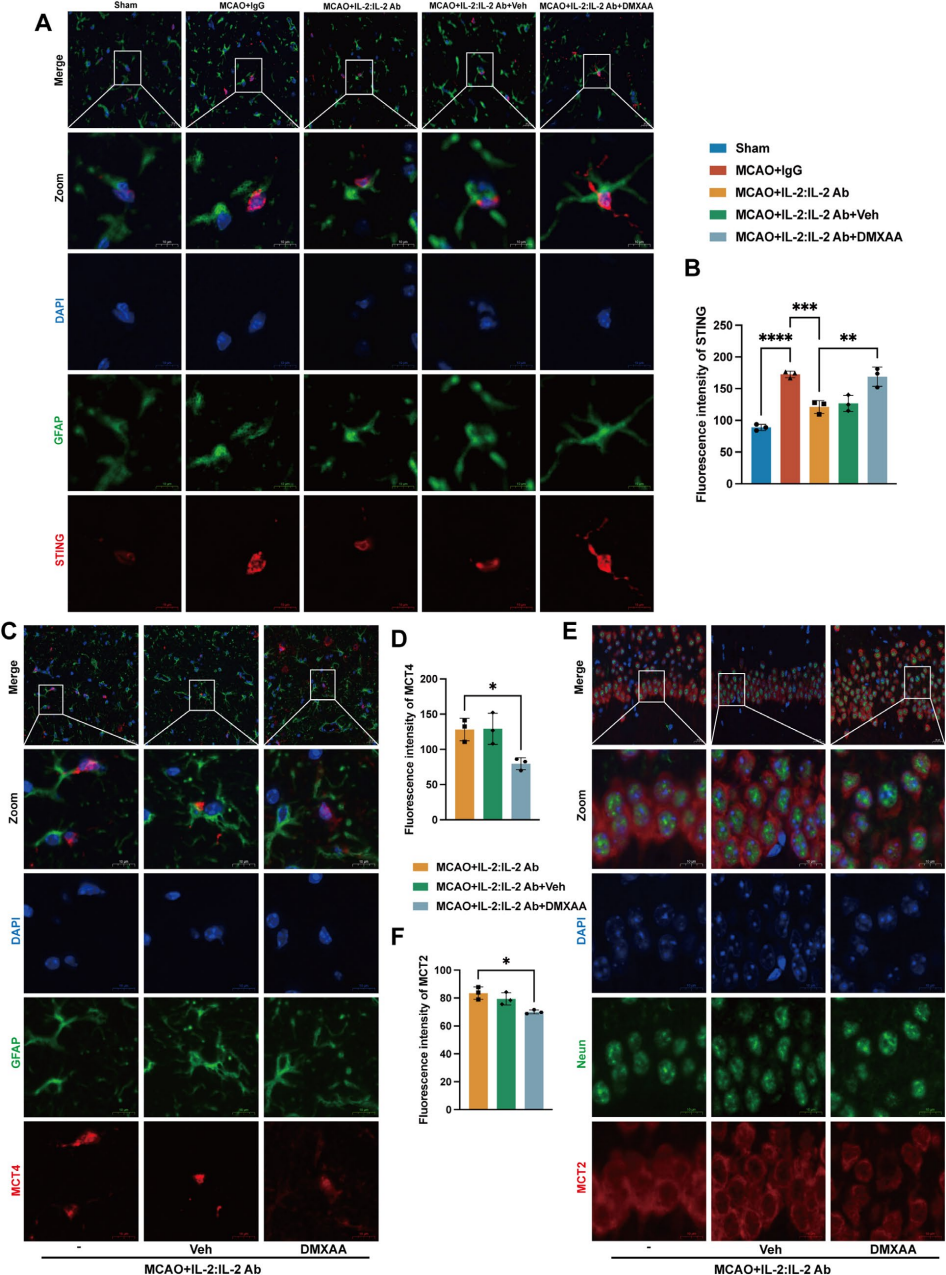
Activation of STING by DMXAA inhibited the enhancement of ANLS by Tregs in mice. Representative images and fluorescence intensity of STING (A–B: F_4,10_ = 34.11, *p* < 0.0001) and MCT4 (C–D: F_2,6_ = 8.882, *p* = 0.0161) in astrocytes and MCT2 (E–F: F_2,6_ = 10.31, *p* = 0.0115) in neurons in the hippocampal CA1 region (*n* = 3). Scale bars = 20 μm and 10 μm. Quantitative data were shown as mean ± SD. *p < 0.05, **p < 0.01, ***p < 0.001, ****p < 0.0001.

The STING‐positive cells in astrocytes in the hippocampal CA1 region were decreased after administration of IL‐2:IL‐2 Ab complex compared with the MCAO + IgG group (Figure 5A,B). The STING activator DMXAA reversed the inhibitory effect of Tregs on STING. In addition, the results of immunofluorescence revealed that DMXAA attenuated the promotion effect of Tregs on MCT (Figure 5C–F). These findings suggested Tregs promoted ANLS by inhibiting STING after stroke.

### 3.6 Activation of STING by DMXAA Suppressed the Enhancement of ANLS by Tregs In Vitro

**FIGURE 6:**
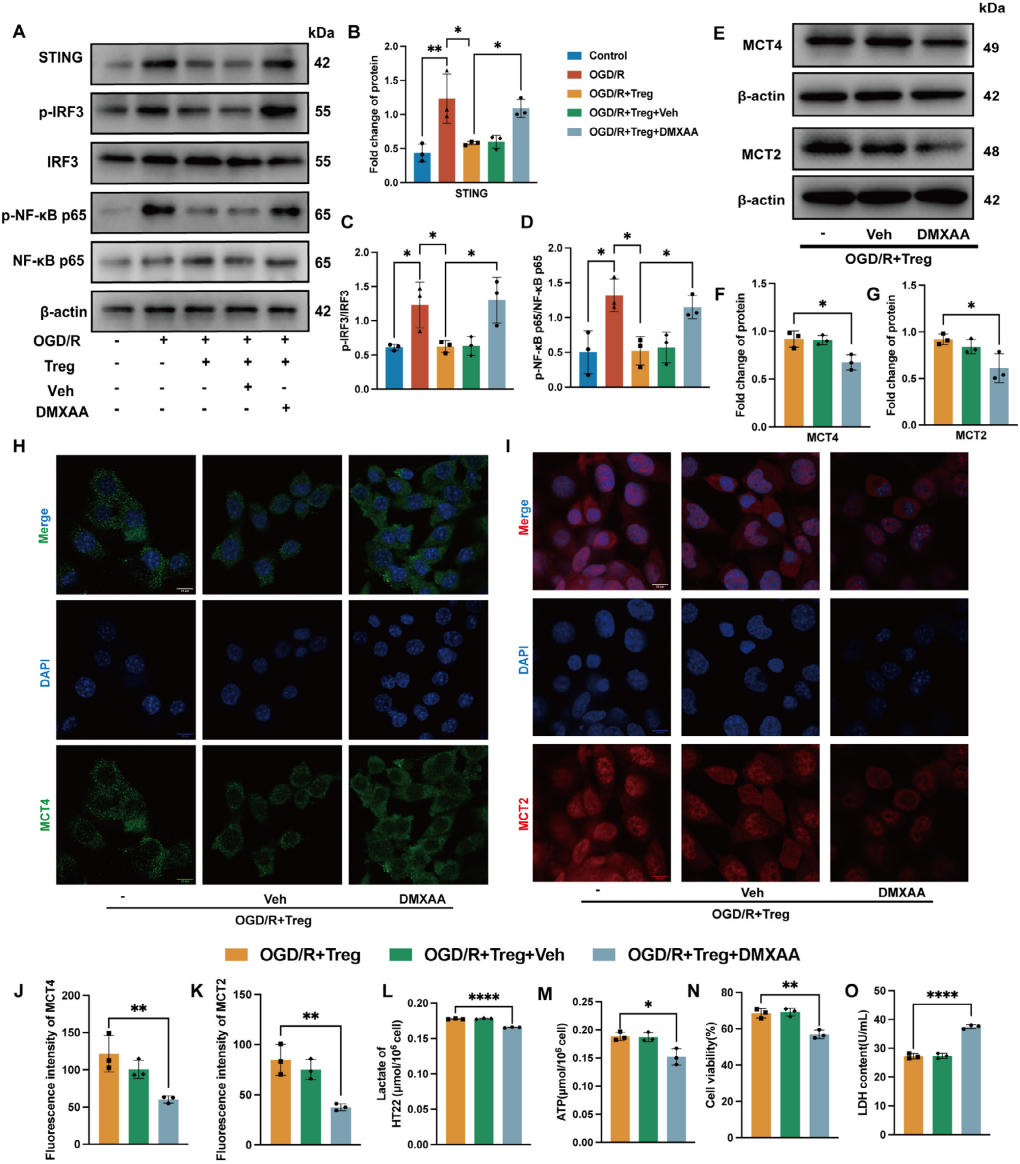
Activation of STING by DMXAA suppressed the enhancement of ANLS by Tregs in vitro. (A) Representative bands of STING, p‐IRF3, IRF3, p‐NF‐κB p65 and NF‐κB p65 of C8‐D1A cells. (B–D) Quantification of the relative protein expression of STING (F_4,10_ = 10.58, *p* = 0.0013), p‐IRF3/IRF3 (F_4,10_ = 7.458, *p* = 0.0047) and p‐NF‐κB p65/NF‐κB p65 (F_4,10_ = 8.502, *p* = 0.0029) (*n* = 3). (E) Representative bands of ANLS‐related proteins MCT4 of C8‐D1A cells, MCT2 of HT22 cells. (F–G) Quantification of the relative protein expression of MCT4 (F_2,6_ = 10.73, *p* = 0.0104) and MCT2 (F_2,6_ = 7.041, *p* = 0.0267) (*n* = 3). (H–K) Immunofluorescence staining images and quantitative analysis of MCT4 (F_2,6_ = 11.24, *p* = 0.0093) in C8‐D1A cells, MCT2 (F_2,6_ = 16.13, *p* = 0.0039) in HT22 cells (*n* = 3). Scale bars = 10 μm. (L) Lactate level of HT22 cells (F_2,6_ = 388.1, *p* < 0.0001) (*n* = 3). (M) ATP level of HT22 cells (F_2,6_ = 11.67, *p* = 0.0086) (*n* = 3). (N) CCK‐8 was used to evaluate cell viability of HT22 cells in different groups (F_2,6_ = 25.56, *p* = 0.0012) (*n* = 3). (O) LDH level of HT22 cells supernatant (F_2,6_ = 152.1, *p* < 0.0001) (*n* = 3). Quantitative data were shown as mean ± SD. *p < 0.05, **p < 0.01, ***p < 0.001, ****p < 0.0001.

The effect of Tregs on STING was further validated in C8‐D1A cells. Western blot analysis showed that Tregs reduced the expression of STING and inhibited the phosphorylation of IRF3 and NF‐κB p65, while DMXAA reversed this effect (Figure 6A–D). As shown in Figure 6E–G, the expression of MCT4 and MCT2 proteins decreased compared with the OGD/R+ Treg group. Moreover, the immunofluorescence intensity of MCT4 and MCT2 in the OGD/R+ Treg group was significantly higher than that in the OGD/R + Treg + DMXAA group (Figure 6H–K). The decrease in lactate and ATP content reflected the declined lactate transport and energy supply in HT22 cells after DMXAA treatment (Figure 6L,M). The results of CCK‐8 and LDH assay showed that DMXAA inhibited the protective effect of Tregs on HT22 cells (Figure 6N,O).

We found that the knockdown of STING (Figure S2A,B) reduced pyroptosis in C8‐D1A cells (Figure S2C–E) and improved the state of astrocytes to promote the production of lactate (Figure S2F–H). Meanwhile, the knockdown of STING (Figure S3A,B) enhanced the expression of MCT4 and MCT2 (Figure S3C,D), thereby increasing the levels of lactate and ATP in neurons and reducing neuronal damage (Figure S3E–H). These data confirmed the cell‐autonomous role of astrocytic STING in this process. In conclusion, Tregs improved the functional state of astrocytes by inhibiting STING, thereby promoting ANLS.

## 4 Discussion

In recent years, immunotherapy has received widespread attention in the treatment of central nervous system diseases. The crosstalk between the central nervous system and the immune system after ischemic stroke is multifaceted. Treg, a key immunomodulator, has been proved to modulate the immune response after stroke, influence multiple cytokine‐mediated inflammation, and attenuate brain damage [34, 35]. In this study, we demonstrated that long‐term repair and neurological recovery of the ischemic brain tissues were improved after enhancement of Tregs with IL‐2:IL‐2 Ab complex. Mechanistically, Tregs improved neurological recovery by promoting ANLS and thereby meeting the energy supply demand required for neuronal remodeling. The application of the STING activator indicated that Tregs promoted ANLS by inhibiting the STING pathway. These findings provide a new entry point to unravel the role of Tregs in stroke.

Tregs exert multifaceted neuroprotective effects by maintaining the homeostasis of the central nervous system. The interaction between Tregs and microglia can create an osteopontin‐enriched microenvironment, which enhances the repair responses of microglia [26]. Meanwhile, Tregs downregulate neurotoxic astrocyte‐related gene expression and inhibit reactive astrogliosis, thereby reducing neuronal apoptosis and alleviating neurological impairment [11]. Furthermore, Tregs promote oligodendrocyte differentiation and myelination to restore white matter integrity [36], and facilitate the delivery of brain‐derived neurotrophic factor (BDNF) to the site of brain injury to support neurological recovery after stroke [37].

The IL‐2:IL‐2 Ab complex is considered a potent enhancer of Tregs, which not only expands the number of Tregs but also enhances the function of Tregs [38]. It has been demonstrated that increasing the number of Tregs by pretreatment with the IL‐2:IL‐2 Ab complex can enhance Treg‐mediated responses and ameliorate ischemic brain injury in the acute phase after stroke [35]. However, the impact on long‐term neurological recovery after stroke remains unclear. There is a delay in the response of Tregs in the adaptive immune response after stroke [39]. Only a small number of Tregs infiltrate into the brain during the acute phase of stroke, whereas a large number of Tregs can be detected in brain tissues in the remote phase after stroke [40, 41]. Our study confirmed that intraperitoneal injection of the IL‐2:IL‐2 Ab complex after stroke increased the amount of Tregs on the 14th day after stroke and reduced neuronal death and myelin loss in the brain tissue on day 21 after stroke. In addition, the expression of synaptic plasticity‐associated proteins Synaptophysin, PSD95, and GAP43 were increased, which helped with the transmission of signals between synapses and improved long‐term motor and memory function. In summary, we found that increasing the number of Tregs improved long‐term neuronal repair and neurological recovery after stroke.

Due to the narrow time window for thrombolytic therapy in acute ischemic stroke patients, only a small proportion benefit from it [3]. Therefore, finding effective interventions that can promote long‐term recovery during the subacute phase has become an important research direction in recent years. In this study, we demonstrated that administration of the IL‐2:IL‐2 Ab complex at 6 h after MCAO expanded the number of Tregs and improved long‐term neurological recovery in stroke mice. Moreover, the clinical safety of the IL‐2:IL‐2 Ab complex has been confirmed in the treatment of autoimmune diseases, hepatitis C vasculitis, and graft‐versus‐host disease [42, 43, 44]. Therefore, our findings provide novel insights and evidence for clinical immunotherapy of subacute stroke patients using IL‐2: IL‐2 Ab complex in the future.

Neurons can efficiently metabolize glucose under physiological conditions and produce ATP through oxidative phosphorylation [45]. However, during the stroke, glucose metabolism is disrupted after neuronal ischemia and hypoxia, and the glycolytic process of lactate becomes an important source of energy supply for neurons. Astrocytes, as the major supplier of lactate, provide energy to neurons by transporting lactate to neurons, promoting neuronal regeneration and long‐term memory consolidation [18, 46]. ANLS depends on MCT, with MCT4 predominantly expressed in astrocytes and MCT2 mainly expressed in neurons [47]. The levels of MCT4 and MCT2 can indirectly reflect the level of ANLS. 4‐CIN, a specific inhibitor of MCT, was shown to attenuate ANLS in previous studies [27]. Our study revealed that the fluorescence intensity of MCT4 and MCT2 was significantly enhanced after increasing Tregs, suggesting that Tregs exerted neuroprotective effects by promoting ANLS. The neuroprotective effect of Tregs on neurons and myelin in the brain tissues was inhibited after the administration of 4‐CIN, and mice showed deterioration of long‐term motor and memory functions and reduced the expression of synaptic plasticity‐associated proteins. These results confirmed that Tregs exerted neuroprotective effects by promoting ANLS following stroke. To clarify the role of Tregs on ANLS, we further examined the expression of MCT4 in C8‐D1A cells and MCT2 in HT22 cells, as well as the content of lactate and ATP in HT22 cells in vitro. The results showed that 4‐CIN attenuated ANLS and reduced the energy supply of lactate and cell viability of HT22 cells when co‐cultured with Tregs. Therefore, we believe that the effect of Tregs on long‐term prognosis of stroke is closely related to the promotion of ANLS.

In addition to serving as an energy substrate, lactate can also act as a signaling molecule to regulate neuronal excitability, synaptic plasticity, and memory consolidation, thereby maintaining neurological homeostasis. Lactate modulates the excitability of hippocampal pyramidal cells [48], and stimulates the expression of synaptic plasticity‐related gene through NMDA receptors and their downstream signaling cascade factors [49]. Lactate can promote hippocampal long duration enhancement and memory consolidation and enhance decision‐making functions in the amygdala and anterior cingulate cortex [46, 50].

The STING signaling pathway, an integral part of the innate immune system, has been widely concerned in cerebral ischemic injury. It has been reported that STING can be activated by any aberrant DNA damage [51]. STING is closely related to the survival and function of astrocytes in central nervous system diseases. In Parkinson's disease, STING promotes astrocyte senescence through the YY1‐LCN2 signaling cascade, exacerbating impairment of motor function [24]. Overactivation of the STING signaling pathway plays a crucial role in NLRP3‐mediated astrocyte pyroptosis in copper‐induced neurotoxicity [23]. Given that astrocytes are the starting point for ANLS, we wondered whether the role of Tregs in promoting ANLS is related to the STING pathway. In our study, we found that Tregs attenuated the immunofluorescence co‐localization of astrocyte and STING in post‐stroke mice. And Tregs decreased the expression of STING and the phosphorylation levels of downstream inflammatory factors IRF3 and NF‐κB in C8‐D1A cells of OGD/R model. These results suggested that Tregs may exert effect by inhibiting the STING pathway in astrocytes after stroke. To further confirm this hypothesis, we employed the STING agonist DMXAA to verify the potential mechanism of STING in the interaction between Tregs and ANLS. In the MCAO mouse model, we observed a decrease in immunofluorescence intensity of MCT4 and MCT2 after activating the STING signaling pathway. In addition, in co‐cultured cells treated with Tregs, DMXAA significantly blocked Treg's promotion of ANLS, reduced energy support, and accelerated HT22 cell death. Collectively, these findings provide compelling evidence that Tregs promote ANLS after stroke by inhibiting the STING pathway, thereby providing energy supply to neurons, attenuating neuronal damage, improving neuronal remodeling, and exerting neuroprotective effects.

Our data demonstrated that Treg expansion was associated with increased levels of TGF‐β and IL‐10. The roles of Treg and STING are quite complex. There is research confirming that Treg‐derived TGF‐β can transcriptionally suppress the mRNA levels of STING, contributing to the decreased MHC expression and elevated PD‐L1 levels [52]. TGF‐β is implicated in the STING‐mediated type I interferon secretion and resultant immune activation, in conjunction with weakened DNA repair capacity and enhanced apoptosis [53]. The cytokines secreted by Tregs are closely related to the expression and function of STING.

There are also some limitations to this study that need to be taken into account. First, in the study of ANLS in mice, this study did not dynamically monitor the migration of lactate in brain tissue. In the future, more accurate lactate tracing technology can be used to improve the detection of brain lactate migration and metabolism [54]. Second, cells co‐cultured in vitro cannot fully mimic the complex interactions that occur in vivo. Although C8‐D1A and HT22 cells are valuable tools for mechanistic studies, they are simplified models that do not fully recapitulate the mature functionality and intricate crosstalk of primary astrocytes and neurons in vivo. Future work utilizing more complex systems, such as primary co‐cultures or organotypic brain slices, will be necessary to confirm and extend our observations. Third, it should be noted that, despite the consistency of our pharmacological data, the potential influence of unknown off‐target effects of DMXAA and 4‐CIN cannot be fully ruled out. Future studies utilizing cell‐specific, conditional STING or MCT knockout models will be required to definitively validate these findings without the complication of pharmacological off‐target effects. Otherwise, STING has been confirmed to mainly exist in astrocytes [55] and microglia [56] within the central nervous system. Whether Tregs suppress STING in astrocytes while also inhibiting STING in microglia in vivo requires further investigation. Given STING's extensive role in cerebral diseases, clarifying this mechanism is crucial for understanding Tregs' action mechanisms and potential side effects. In addition, in order to minimize the influence of variables on mechanism research, this study only included male C57BL/6 mice, ignoring the role of gender and strain‐specificity in the pathological mechanisms of cerebral diseases. Genetic and gender backgrounds may influence the pathophysiology and prognosis of ischemic stroke. Finally, although the MCAO model is standard, inherent variability may still exist, such as the inherent variability in infarct volume due to differences in the exact location of filaments and the impact of anesthesia on brain metabolism, which may also affect experimental results.

## 5 Conclusions

In this study, we demonstrated that Tregs alleviated cerebral injury in the remote phase after stroke, enhanced neuroplasticity, and promoted the recovery of motor and cognitive functions. Furthermore, we revealed a novel mechanism by which Tregs promoted ANLS through inhibiting the STING pathway in astrocytes, thereby providing energy supply for neuronal repair after stroke. These findings provide a new theoretical basis for the use of Tregs as an immunotherapy for the treatment of stroke.

## Author Contributions

Y.M.: conceptualization, data curation, formal analysis, methodology, visualization, writing – original draft. X.L., Y.B., P.D.: formal analysis, methodology. Z.J., L.L., W.F., H.L.: methodology, supervision. X.Z., K.Z.: formal analysis. J.C., L.L., Y.X.: writing – original draft. M.Y., M.C., Y.Y.: visualization. B.Z.: funding acquisition, supervision, writing – review and editing. All authors read and approved the final manuscript.

## Funding

This study was supported by the National Natural Science Foundation of China (82072129).

## Ethics Statement

The animal study was carried out by a protocol approved by the Animal Experiment Center of the Second Affiliated Hospital of Harbin Medical University. The experimental protocol was approved by the Ethics Committee (Approval No.: YJSDW2024‐009).

## Conflicts of Interest

The authors declare no conflicts of interest.

## Supporting information


**Figure S1:** The purity of Tregs and the proportion of their coculture with C8‐D1A cells in vitro. (A) Representative flow cytometry plots and the percentage of CD4^+^CD25^+^Foxp3^+^ cells. (B) C8‐D1A cells were cocultured with Tregs in various proportions, and CCK‐8 was used to evaluate the cell viability of C8‐D1A cells.
**Figure S2:** The knockdown of STING reduces pyroptosis in C8‐D1A cells, enhancing their survival and lactate production. (A–B) The expression and quantification of STING following the successful knockdown of the si‐STING in C8‐D1A cells. (F_2,6_ = 11.51, *p* = 0.0088) (*n*=3). (C) Representative bands of pyroptosis‐related proteins NLRP3 and GSDMD‐N of C8‐D1A cells. (D–E) Quantification of the relative protein expression of NLRP3 (F_2,6_ = 11.11, *p* = 0.0096) and GSDMD‐N (F_2,6_ = 12.89, *p* = 0.0067) (*n* = 3). (F) CCK‐8 was used to evaluate cell viability of C8‐D1A cells in different groups (F_2,6_ = 13.45, *p* = 0.0061) (*n* = 3). (G) LDH level of C8‐D1A cells supernatant (F_2,6_ = 18.88, *p* = 0.0026) (*n* = 3). (H) Lactate level of C8‐D1A cells (F_2,6_ = 22.03, *p* = 0.0017) (*n* = 3). Quantitative data were shown as mean ± SD. *p < 0.05, **p < 0.01.
**Figure S3:** The knockdown of STING mimics the effects of Tregs on ANLS and neuroprotection in vitro. (A) Representative bands of STING and MCT4 of C8‐D1A cells, MCT2 of HT22 cells. (B–D) Quantification of the relative protein expression of STING (F _2,6_ = 14.53, *p* = 0.0050), MCT4 (F _2,6_ = 14.77, *p* = 0.0048) and MCT2 (F_2,6_ = 9.990, *p* = 0.0123) (*n* = 3). (E) Lactate level of HT22 cells (F_2,6_ = 5.774, *p* = 0.0400) (*n* = 3). (F) ATP level of HT22 cells (F_2,6_ = 34.38, *p* = 0.0005) (*n* = 3). (G) CCK‐8 was used to evaluate cell viability of HT22 cells in different groups (F_2,6_ = 46.42, *p* = 0.0002) (*n* = 3). (H) LDH level of HT22 cells supernatant (F_2,6_ = 13.64, *p* = 0.0059) (*n* = 3). Quantitative data were shown as mean ± SD. *p < 0.05, **p < 0.01, ***p < 0.001.

## Data Availability

The data that support the findings of this study are available from the corresponding author upon reasonable request.
