# Supplementary material for: Tregs Promote Astrocyte‐Neuron Lactate Shuttle via Inhibiting STING Pathway to Improve Neurological Recovery After Ischemic Stroke
Source: CNS Neurosci Ther. 2026 Jan 19;32(1):e70753. doi: 10.1002/cns.70753 (PMC12813866; doi:10.1002/cns.70753)
Supplement: Supplementary file 1 — Figure S1: The purity of Tregs and the proportion of their coculture with C8‐D1A cells in vitro. (A) Representative flow cytometry plots and the percentage of CD4+CD25+Foxp3+ cells. (B) C8‐D1A cells were cocultured with Tregs in various proportions, and CCK‐8 was used to evaluate the cell viability of C8‐D1A cells. Figure S2: The knockdown of STING reduces pyroptosis in C8‐D1A cells, enhancing their survival and lactate production. (A–B) The expression and quantification of STING following the successful knockdown of the si‐STING in C8‐D1A cells. (F2,6 = 11.51, p = 0.0088) (n=3). (C) Representative bands of pyroptosis‐related proteins NLRP3 and GSDMD‐N of C8‐D1A cells. (D–E) Quantification of the relative protein expression of NLRP3 (F2,6 = 11.11, p = 0.0096) and GSDMD‐N (F2,6 = 12.89, p = 0.0067) (n = 3). (F) CCK‐8 was used to evaluate cell viability of C8‐D1A cells in different groups (F2,6 = 13.45, p = 0.0061) (n = 3). (G) LDH level of C8‐D1A cells supernatant (F2,6 = 18.88, p = 0.0026) (n = 3). (H) Lactate level of C8‐D1A cells (F2,6 = 22.03, p = 0.0017) (n = 3). Quantitative data were shown as mean ± SD. *p < 0.05, **p < 0.01. Figure S3: The knockdown of STING mimics the effects of Tregs on ANLS and neuroprotection in vitro. (A) Representative bands of STING and MCT4 of C8‐D1A cells, MCT2 of HT22 cells. (B–D) Quantification of the relative protein expression of STING (F 2,6 = 14.53, p = 0.0050), MCT4 (F 2,6 = 14.77, p = 0.0048) and MCT2 (F2,6 = 9.990, p = 0.0123) (n = 3). (E) Lactate level of HT22 cells (F2,6 = 5.774, p = 0.0400) (n = 3). (F) ATP level of HT22 cells (F2,6 = 34.38, p = 0.0005) (n = 3). (G) CCK‐8 was used to evaluate cell viability of HT22 cells in different groups (F2,6 = 46.42, p = 0.0002) (n = 3). (H) LDH level of HT22 cells supernatant (F2,6 = 13.64, p = 0.0059) (n = 3). Quantitative data were shown as mean ± SD. *p < 0.05, **p < 0.01, ***p < 0.001. [file CNS-32-e70753-s001.doc]

Supplementary material

**Tregs promote astrocyte-neuron lactate shuttle via** **inhibiting STING pathway to improve neurological recovery after ischemic stroke**

Yao Meng, Xiaoyan Li, Yonghong Bi, Pengyu Duan, Zhehao Jin, Lan Luo, Weiyu Feng, Hangbing Li, Xiangcheng Zhao, Kun Zuo, Jiali Chen, Longfei Li, Yuling Xing, Miao Yu, Muyan Cui, Yang Yu, Bing Zhang^*^


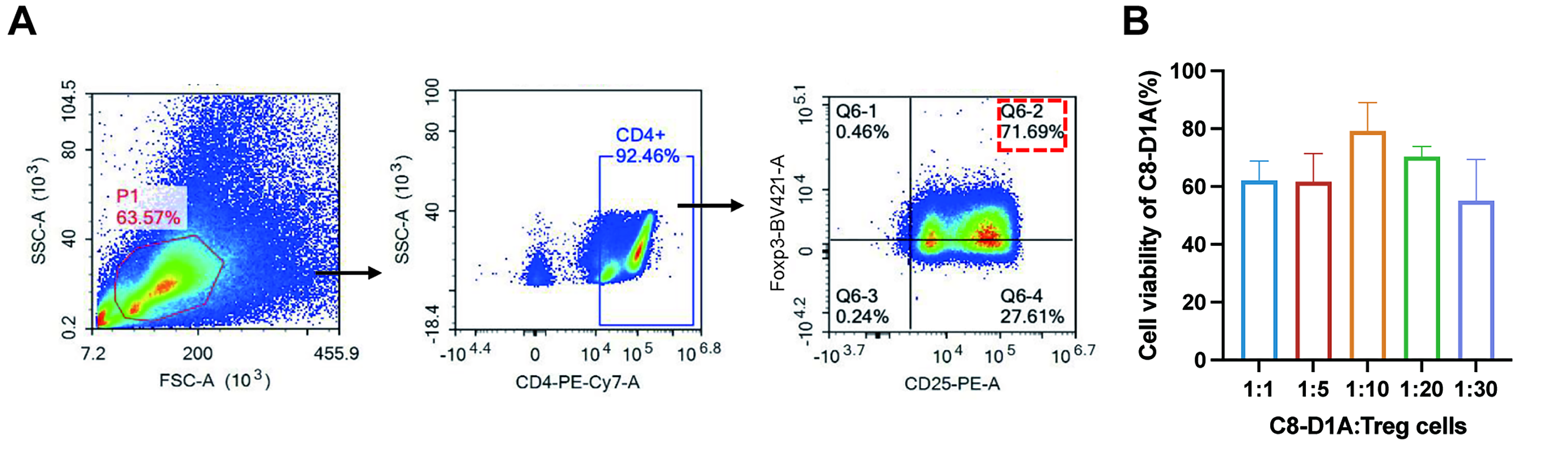


**Fig. S1.** **The purity of Tregs and the proportion of their coculture with C8-D1A cells *in vitro*.** (A) Representative flow cytometry plots and the percentage of CD4^+^CD25^+^Foxp3^+^ cells. (B) C8-D1A cells were cocultured with Tregs in various proportions, and CCK-8 was used to evaluate the cell viability of C8-D1A cells.

**
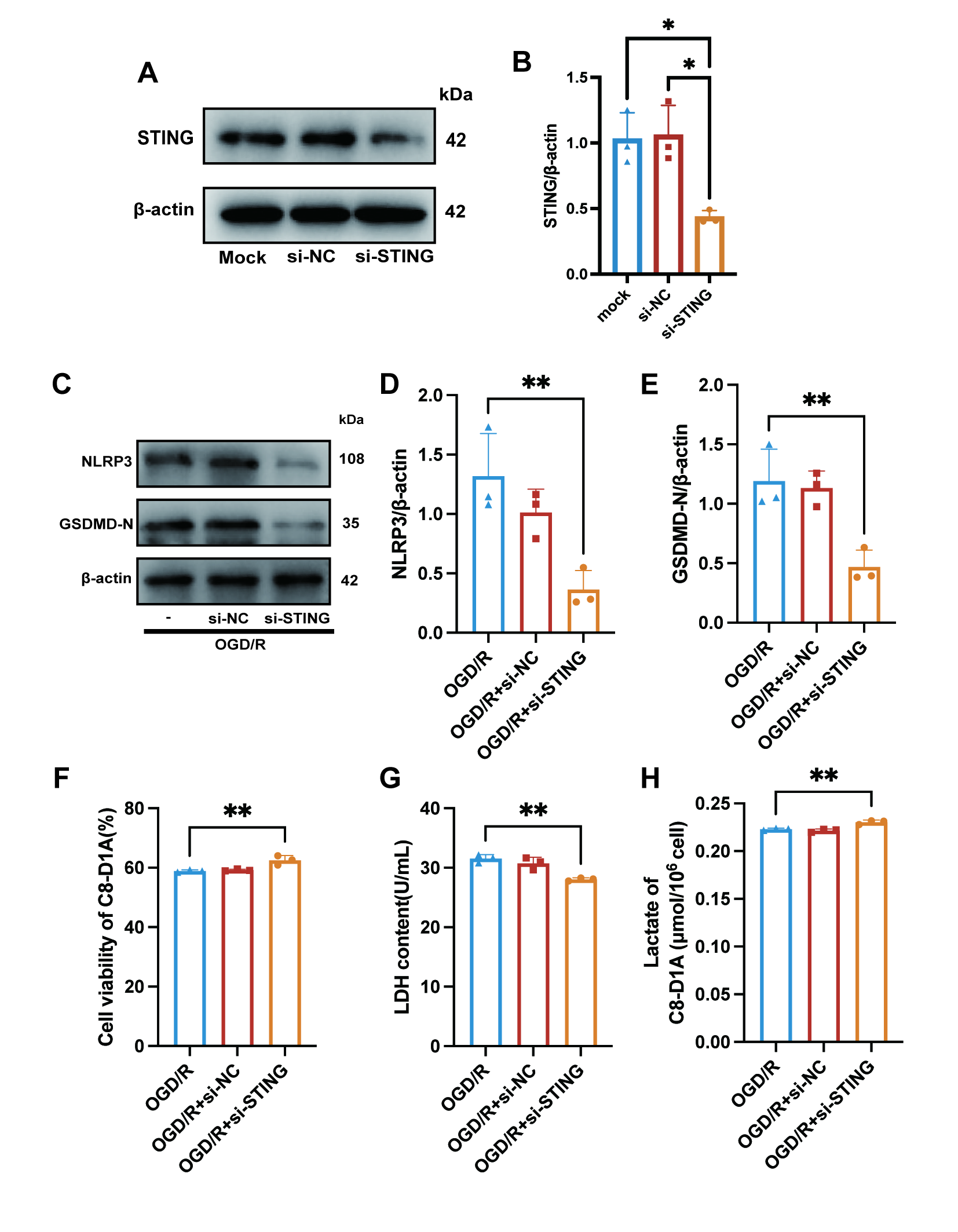
**

**Fig. S2. The knockdown of STING reduces pyroptosis in C8-D1A cells, enhancing their survival and lactate production**. (A-B) The expression and quantification of STING following the successful knockdown of the si-STING in C8-D1A cells. (F_2,6_ = 11.51, *P* = 0.0088). (C) Representative bands of pyroptosis-related proteins NLRP3 and GSDMD-N of C8-D1A cells. (D-E) Quantification of the relative protein expression of NLRP3 (F_2,6_ = 11.11, *P* = 0.0096) and GSDMD-N (F_2,6_ = 12.89, *P* = 0.0067) (n = 3). (F) CCK-8 was used to evaluate cell viability of C8-D1A cells in different groups (F_2,6_ = 13.45, *P* = 0.0061) (n = 3). (G) LDH level of C8-D1A cells supernatant (F_2,6_ = 18.88, *P* = 0.0026) (n = 3). (H) Lactate level of C8-D1A cells (F_2,6_ = 22.03, *P* = 0.0017) (n = 3). Quantitative data were shown as mean ± SD. ^*^*P* < 0.05, ^**^*P* < 0.01.

**
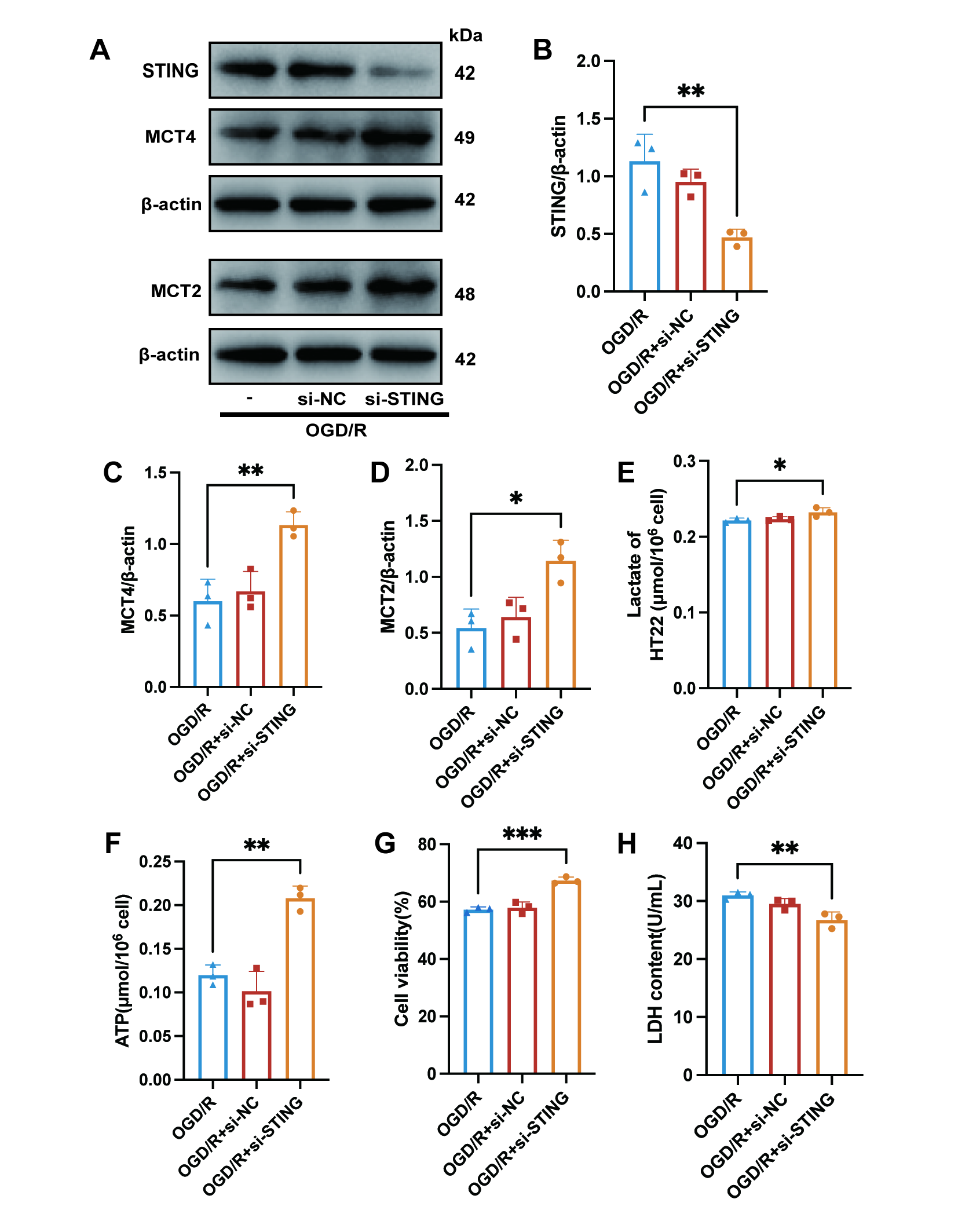
**

**Fig. S3. The knockdown of STING mimics the effects of Tregs on ANLS and neuroprotection *in vitro*.** (A) Representative bands of STING and MCT4 of C8-D1A cells, MCT2 of HT22 cells. (B-D) Quantification of the relative protein expression of STING (F _2,6_ = 14.53, *P* = 0.0050), MCT4 (F _2,6_ = 14.77, *P* = 0.0048) and MCT2 (F_2,6_ = 9.990, *P* = 0.0123) (n = 3). (E) Lactate level of HT22 cells (F_2,6_ = 5.774, *P* = 0.0400) (n = 3). (F) ATP level of HT22 cells (F_2,6_ = 34.38, *P* = 0.0005) (n = 3). (G) CCK-8 was used to evaluate cell viability of HT22 cells in different groups (F_2,6_ = 46.42, *P* = 0.0002) (n = 3). (H) LDH level of HT22 cells supernatant (F_2,6_ = 13.64, *P* = 0.0059) (n = 3). Quantitative data were shown as mean ± SD. ^*^*P* < 0.05, ^**^*P* < 0.01, ^***^*P* < 0.001.
